# Intrathecal combined with intravenous eravacycline for the treatment of multisite carbapenem-resistant *Acinetobacter baumannii* infections (intracranial, pulmonary, and bloodstream) in a post-trauma adolescent female: a case report

**DOI:** 10.3389/fmed.2026.1829527

**Published:** 2026-05-25

**Authors:** Jieluan Lu, Junjie Zhong, Wenbing Qiu, Qian Zhang

**Affiliations:** Department of Pharmacy, The First Affiliated Hospital of Shantou University Medical College, Shantou, Guangdong, China

**Keywords:** adolescent, carbapenem-resistant *Acinetobacter baumannii* (CRAB), central nervous system (CNS) infections, eravacycline, intrathecal administration

## Abstract

**Background:**

Carbapenem-resistant *Acinetobacter baumannii* (CRAB) is a leading cause of hospital-acquired infection among critically ill patients, with extremely limited therapeutic options, particularly for central nervous system (CNS) infections. Eravacyline, a novel fully synthetic fluorocycline, demonstrates potent *in vitro* activity against CRAB but exhibits poor penetration across the blood–brain barrier (BBB).

**Case presentation:**

A 17-years-old female with severe traumatic brain injury developed concurrent intracranial, pulmonary, and bloodstream CRAB infections. Initial systemic antimicrobial therapy, including intravenous colistin and eravacycline, failed to control the intracranial infection. After switching to a regimen incorporating intrathecal eravacycline (initial dose 2 mg, followed by 5 mg daily) combined with high-dose intravenous cefoperazone-sulbactam and nebulized colistin, the patient showed rapid clinical and microbiological improvement. Serial cerebrospinal fluid (CSF) metagenomic next-generation sequencing (mNGS) revealed a dramatic reduction in pathogen load, with eventual eradication of CRAB.

**Conclusion:**

This case highlights the potential role of intrathecal eravacycline as a salvage therapy for CRAB meningitis, particularly in cases of multifocal, extensively drug-resistant infection. Further pharmacokinetic and safety studies are warranted to optimize its use in CNS infections.

## 1 Introduction

Traumatic brain injury (TBI) remains a leading cause of mortality and long-term disability among young individuals worldwide. Post-neurosurgical central nervous system infection (CNSI) is a devastating complication, with reported incidence ranging from 4.6% to 25% (1) and associated mortality rates of 3%–33% (2). Survivors often suffer from permanent neurological deficits even after clinical cure.

Carbapenem-resistant *Acinetobacter baumannii* (CRAB) has emerged as a dominant multidrug-resistant pathogen in intensive care units (ICUs), characterized by remarkable environmental persistence and extensive drug resistance. CRAB frequently causes hospital-acquired pneumonia (HAP) and bloodstream infections (BSI), with mortality rates ranging from 43% to 75% (3–6). According to 2024 CHINET surveillance data in China, *A. baumannii* ranks fifth among clinical isolates, with carbapenem resistance rates exceeding 76%. Therapeutic options for CRAB are severely limited, and CNS invasion presents an even greater therapeutic challenge.

Effective treatment of CRAB CNS infections is hampered by two key factors: poor penetration of most antibiotics across the BBB and elevated minimum inhibitory concentrations (MICs) due to bacterial resistance. Consequently, intravenous therapy alone is often insufficient to achieve therapeutic CSF concentrations, necessitating alternative delivery strategies such as intrathecal administration.

Herein, we report a case of severe TBI complicated by multi-site (intracranial, pulmonary, and bloodstream) CRAB infections that was successfully treated with a regimen including intrathecal eravacycline, a novel fluorinated tetracycline agent.

## 2 Case presentation

### 2.1 Presentation and surgery

A 17-years-old female was admitted on October 1, 2025, following a motorcycle accident resulting in cerebral hemorrhage and skull fracture. On admission, she was comatose (Glasgow Coma Scale score 6) with bilateral mydriasis (4 mm). Emergency neurosurgical procedures were performed immediately, including evacuation of bilateral epidural, subdural, and intracerebral hematomas; bilateral decompressive craniectomy; dural augmentation; and placement of an intraparenchymal intracranial pressure monitor. Bilateral epidural drains were also inserted during the same operation. Postoperatively, she was admitted to the ICU and placed on mechanical ventilation. Empirical intravenous antimicrobial therapy with meropenem (1 g every 8 h) was initiated in accordance with 2016 Infectious Diseases Society of America (IDSA)/American Thoracic Society (ATS) guidelines for empirical treatment of hospital-acquired pneumonia (HAP) or ventilator-associated pneumonia (VAP) in patients at high risk of multidrug-resistant Gram-negative bacilli (7).

The patient was a previously healthy 17-years-old adolescent female with no notable chronic medical illnesses, including respiratory, cardiovascular, hepatic, renal, neurologic, or endocrine disorders. She had no history of prior surgery, blood transfusion, or long-term medication use. No known drug allergies were documented. Family history was unremarkable for hereditary diseases, immunodeficiency, or CNS disorders. Psychosocial history revealed no history of smoking, alcohol consumption, or other high-risk behaviors. All clinical information was obtained from the medical records and the patient’s legal guardian.

### 2.2 Development and diagnosis of infection

Bilateral epidural drains were removed on day 5 per routine neurosurgical protocol. Sputum culture grew *Staphylococcus aureus* (*S. aureus*), with susceptibility testing showing resistance only to penicillin G and susceptibility to all other tested agents. On day 8, bronchoalveolar lavage fluid culture also grew *Staphylococcus aureus* with an identical susceptibility profile.

On postoperative day 10, the patient developed a high-grade fever (39 °C). Inflammatory markers were elevated: white blood cell count (WBC) 14.03 × 10^9^/L, neutrophils 91.3%, C-reactive protein (CRP) 63.7 mg/L, and procalcitonin (PCT) 1.00 ng/mL. Initial sputum metagenomic next-generation sequencing (mNGS) suggested influenza A virus and *S. aureus*, prompting antiviral therapy.

By day 12, CSF analysis confirmed severe infection: turbid orange fluid, glucose < 0.12 mmol/L, and nucleated cell count 12,160 × 106/L. Blood mNGS detected *Klebsiella pneumoniae* and *A. baumannii*. Head CT showed increased subdural effusion. Given the patient’s persistent fever unresponsive to broad-spectrum antibiotics and marked CSF derangements, a diagnosis of CNS infection was strongly suspected. In accordance with the 2017 IDSA guideline for healthcare-associated ventriculitis and meningitis, the meropenem dosage was escalated to 2 g every 8 h to achieve adequate CSF concentrations for susceptible Gram-negative CNS pathogens (8, 9). Vancomycin (1 g every 8 h, intravenous pump infusion), and polymyxin B (750,000 U every 12 h, intravenous pump infusion; total daily 1.5 million units) were added to cover the identified pathogens.

Despite these interventions, fever persisted. Subsequently, sputum culture performed on day 14 confirmed the presence of CRAB. Antimicrobial susceptibility testing (AST) revealed susceptibility to minocycline (MIC = 4 mg/L) and tigecycline (MIC = 1 mg/L), intermediate susceptibility to colistin (MIC ≤ 0.5 mg/L) and cefoperazone-sulbactam (MIC = 32 mg/L), and resistance to all other tested agents. Susceptibility breakpoints were interpreted according to the guidelines of the Clinical and Laboratory Standards Institute (CLSI), except for tigecycline and cefoperazone-sulbactam, which followed the European Committee on Antimicrobial Susceptibility Testing (EUCAST) criteria. CSF mNGS definitively identified *A. baumannii* carrying the OXA-23 resistance gene (4,252 sequences, 82.95% abundance). The high sequence read count of OXA 23-positive CRAB in CSF was supportive of clinically significant infection rather than colonization, indicating concurrent intracranial CRAB infection. However, owing to the poor BBB penetration of most anti-CRAB agents, initial systemic therapy failed to eradicate the intracranial infection. The persistently high CSF pathogen load, clinical deterioration, and the need to achieve direct therapeutic concentrations within the CNS prompted a critical shift in management strategy.

### 2.3 Adjusted antimicrobial therapy and clinical course

Given the multisite CRAB infection, the regimen was revised on day 14 to target CRAB specifically: intravenous colistimethate sodium (CMS) (75 mg every 12 h) plus intrathecal CMS (5 mg daily) combined with intravenous eravacycline (50 mg every 12 h). On day 17, a lumbar cistern drainage catheter was placed for management of intracranial hypertension and to facilitate CSF sampling and subsequent intrathecal injections via the drain.

**FIGURE 1:**
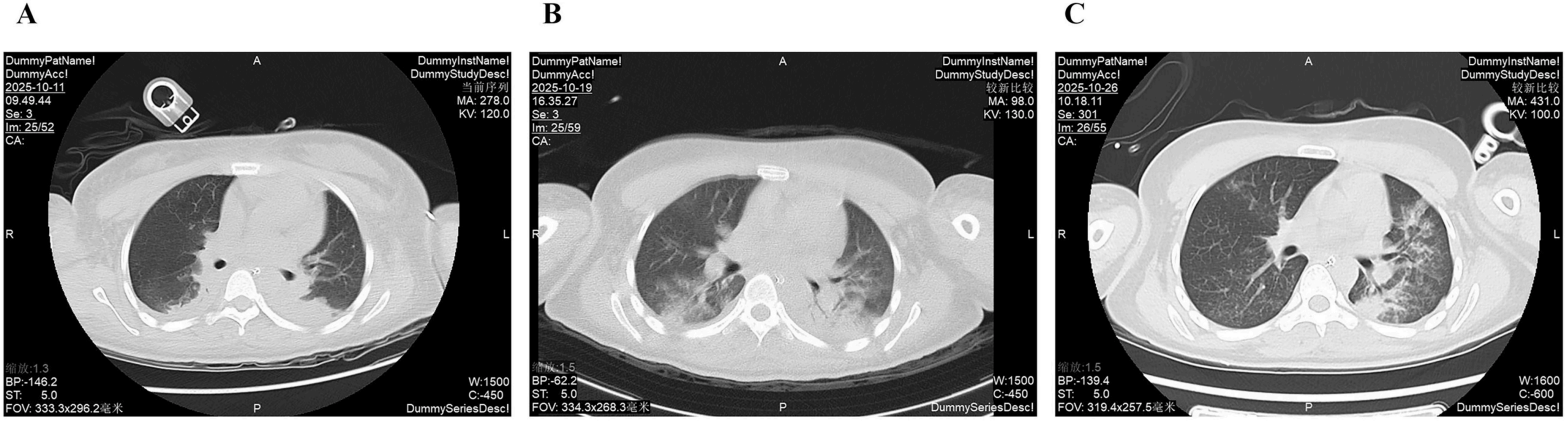
Chest CT images of the patient’s on Day 11 **(A)**, Day 19 **(B)**, Day 26 **(C)**.

Between day 14 and day 23, the patient showed progressive improvement in systemic and pulmonary infection control. Systemic inflammatory markers declined markedly: PCT decreased from 1.16 ng/mL to 0.11 ng/mL, CRP from 81.60 mg/L to 14.70 mg/L, and WBC from 17.80 × 10^9^/L to 14.68 × 10^9^/L. Concurrently, CSF parameters improved substantially: nucleated cell count decreased from 12,160 × 10^6^/L to 80 × 10^6^/L and protein from 4.09 g/L to 1.45 g/L, indicating a favorable response of intracranial infection to antimicrobial therapy. Chest CT performed on day 26 revealed significant resolution of bilateral pulmonary infiltrates and near-complete absorption of pleural effusions (Figure 1), confirming effective control of pulmonary infection. The intraparenchymal intracranial pressure monitor was maintained until day 25. Despite these improvements, the patient remained comatose with intermittent fever, and CSF mNGS on day 24 still showed a high burden of *A. baumannii* (11,794 sequences), indicating persistent intracranial infection requiring further regimen optimization.

Therefore, on day 27, a second major adjustment to the antimicrobial regimen was implemented. Intravenous and intrathecal CMS was discontinued, and therapy was switched to intravenous plus intrathecal eravacycline. In the absence of established dosing guidelines for intrathecal eravacycline in adolescents, and with reference to published experience with intrathecal tigecycline and pharmacokinetic safety considerations, a starting dose of 2 mg was used to minimize the risk of neurotoxicity. After confirming favorable tolerability and persistent intracranial infection, the dose was escalated to 5 mg daily.

For intrathecal administration, 50 mg of eravacycline lyophilized powder was dissolved in 5 mL of preservative-free 0.9% sodium chloride injection to achieve a concentration of 10 mg/mL, with further dilution performed according to the intended intrathecal dose. Each intrathecal injection was administered slowly over 3–5 min, followed by a flush with 3–5 mL of preservative-free normal saline. Administration was given once daily with close neurological monitoring throughout the treatment course. The regimen was well tolerated in this adolescent patient.

In addition, high-dose intravenous cefoperazone–sulbactam (1:1 ratio, 2 g every 4 h by continuous intravenous pump infusion) and nebulized CMS (75 mg every 8 h) were administered concurrently. This combination regimen was designed to target multidrug-resistant infections involving both the pulmonary and intracranial compartments.

### 2.4 Clinical improvement and outcome

**FIGURE 2:**
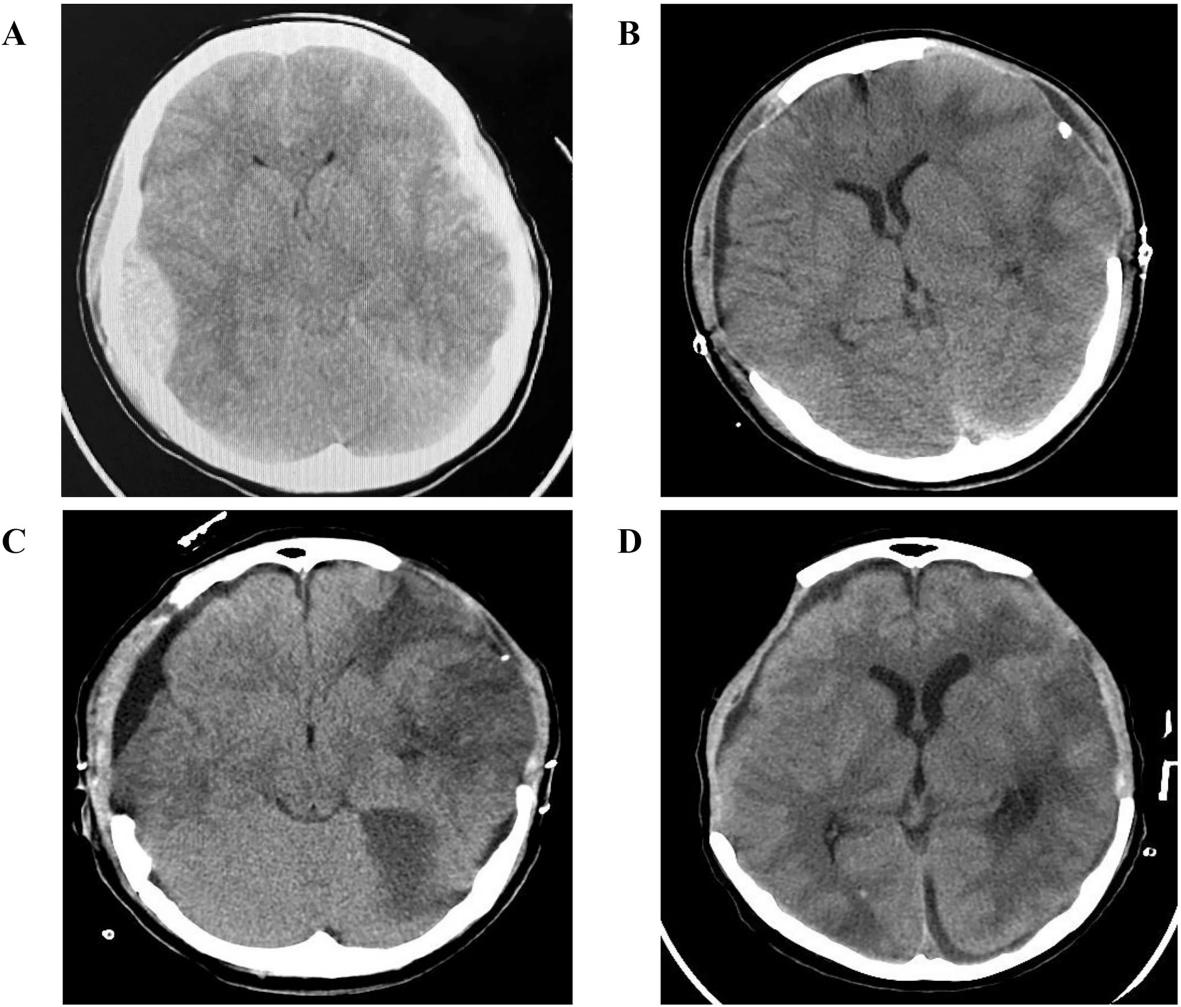
Head CT images of the patient’s on Day 1 **(A)**, Day 11 **(B)**, Day 19 **(C)**, and Day 26 **(D)**.

Following initiation of intrathecal eravacycline, the patient’s neurological status improved progressively, and head CT demonstrated evolution into the recovery phase (Figure 2). By day 36, the patient had regained consciousness and was able to follow commands. Serial CSF mNGS monitoring revealed a marked reduction in pathogen load: from 11,794 sequence reads (day 24) to 79 reads (day 34), and ultimately to undetectable levels (day 60) (Table 1). Systemic inflammatory markers gradually normalized (Table 2).

**TABLE 1:** Cerebrospinal fluid (CSF) test results.

Date	Color	Transparency	Protein (g/L)	Glucose (mmol/L)	Chloride (mmol/L)	Leukocyte count (cells/mm^3^)	mNGS
10.12	Orange	Turbid	4.09	<0.12	111.7	12160	NA
10.15	Orange-yellow	Turbid	3.75	0.57	114.2	1770	*A. baumannii*^a^: 4252 sequence
10.22	Hazel	Slightly turbid	1.45	4.1	115.6	80	NA
10.26	Brown	Slightly turbid	3.34	4.84	119.2	3	*A. baumannii*^a^: 11794 sequence
11.4	NA						*A. baumannii*: 74 sequence
11.10	Yellow	Transparent	2.41	4.14	115	68	NA
11.15	NA						*A. baumannii*: 75 sequence
11.29	NA						Negative

NA, not available.

*^a^*Antimicrobial resistance genes blaoxA-23 were detected.

**TABLE 2:** Infection indicators.

Date	T (°C)	WBC (10 × 10∧9/L)	NEU (%)	CRP (mg/L)	PCT (ng/mL)
10.10	39	14.03	91.30	42.1	1
10.12	38	10.25	92.1	42.1	0.07
10.14	38.3	19.31	94.9	41.6	0.51
10.15	38.3	17.35	94.8	35.1	0.56
10.22	38.7	8.01	77.3	6.6	0.05
10.24	37.2	14.68	94.6	14.7	NA
10.26	37.5	9.72	95.6	9.8	0.11
10.27	38.2	13.86	92.5	6.6	NA
11.1	37.6	9.73	86.1	40.9	1.68
11.4	37.2	9.54	86	10.8	1.67
11.7	37.5	9	85.6	40.1	2.02
11.10	37	4.21	69.8	1.8	0.92

NA, not available.

The lumbar drainage catheter was removed on day 34. Intravenous and intrathecal eravacycline were discontinued on day 38, and oral trimethoprim-sulfamethoxazole was initiated to facilitate step-down from intravenous to oral antimicrobial therapy for rehabilitation. Owing to its favorable oral bioavailability, satisfactory BBB penetration, and *in vitro* activity against CRAB, trimethoprim-sulfamethoxazole was used for sequential suppressive therapy following CSF sterilization. Intravenous cefoperazone–sulbactam was continued concurrently until day 56. On day 41, the patient was transferred to a general ward for rehabilitation with full consciousness and good cooperation.

## 3 Discussion

Eravacycline, a novel fully synthetic fluorocycline, exhibits potent *in vitro* activity against CRAB, with a reported MIC_90_ of 1 μg/mL against a global collection of isolates (10). Comparative studies suggest that this fluorocycline may demonstrate higher *in vitro* potency than tigecycline against *A. baumannii*, including strains with tigecycline resistance (11). This superior potency provided a pharmacologic rationale for its use in this case after failure of prior regimens. A direct clinical comparison remains under investigation; a randomized controlled trial protocol comparing eravacycline and tigecycline for complicated intra-abdominal infections reported all-cause mortality rates of 17.7% and 18.7%, respectively, in sample size calculations, highlighting ongoing efforts to delineate their comparative clinical efficacy (11).

However, like other tetracycline analogs, it demonstrates poor penetration across the intact BBB. Both tigecycline and eravacycline achieve low CSF concentrations when administered intravenously, limiting their efficacy for meningitis or ventriculitis. Clinical evidence supporting direct CNS delivery largely exists for tigecycline, with case reports and small case series documenting its intrathecal use for multi-drug-resistant Gram-negative meningitis (12). A recent retrospective cohort study further supports this strategy, showing that intravenous plus intrathecal/intraventricular tigecycline resulted in a significantly higher microbiological clearance rate (83.33%) compared with intravenous therapy alone (22.22%) in post-neurosurgical CRAB intracranial infections (13). While clinical outcomes may vary, with one case series reporting a 30-days survival rate of 25% despite a 66% microbiological response rate (12), the principle of direct CNS delivery to overcome pharmacological barriers is well-established. These reports indicate favorable clinical and microbiological responses with acceptable tolerability, supporting the feasibility of direct CNS administration of tetracycline derivatives.

Pharmacokinetic studies demonstrate that intravenous eravacycline achieves only minimal CSF concentrations (14), which are subtherapeutic for meningitis. This pharmacokinetic limitation underscores the necessity for alternative routes such as intrathecal delivery to achieve effective CSF concentrations in patients with meningitis (15, 16). Due to insufficient clinical evidence, the IDSA recommends cautious selection of eravacycline only when alternative options have failed. To our knowledge, this is the first documented case of intrathecal eravacycline use for CRAB meningitis in an adolescent with post-traumatic multisite infection. Given the absence of pediatric data, we exercised caution by initiating therapy at a low dose of 2 mg, closely monitoring for adverse effects and tolerability. After confirming safety, the dose was escalated to 5 mg daily, which resulted in satisfactory clinical outcomes. Eravacycline, with its unique halogenated structure and favorable resistance profile against some tetracycline-resistant strains, offers a promising new option. This case thus expands the limited salvage armamentarium for the treatment of challenging neurosurgical infections.

Pharmacokinetic studies in patients with ventilator-associated pneumonia demonstrate that nebulized CMS achieves epithelial lining fluid concentrations several hundred-fold higher than concurrent plasma levels, creating a substantial concentration gradient at the alveolar surface (17). From a clinical perspective, studies in critically ill patients with CRAB pneumonia suggest that regimens incorporating nebulized colistin, as an adjunct to intravenous therapy, may be associated with improved outcomes (18). The favorable safety profile of the inhaled route was an significant consideration within the complex multidrug therapy for this multifocal infection. By targeting drug delivery to the lungs, nebulization avoids high systemic drug levels that are the main driver of the nephrotoxicity and neurotoxicity commonly associated with intravenous polymyxin therapy (17). A retrospective observational study of patients receiving nebulized colistin for multidrug-resistant pneumonia reported no incidents of nephrotoxicity or neurotoxicity directly attributable to the inhaled therapy (19). This favorable safety profile was critical in the present case, allowing aggressive local therapy for pulmonary infection without increasing cumulative toxicity from concurrent systemic agents targeting intracranial and bloodstream infections.

Sulbactam, a β-lactamase inhibitor, exerts intrinsic bactericidal activity against *A. baumannii* binding to penicillin-binding proteins, particularly PBP1 and PBP3. This unique dual mechanism underpins its continued clinical utility despite widespread resistance to other β-lactam agents (20). Current international guidelines, including those from the IDSA, recommend high-dose ampicillin-sulbactam as a preferred or alternative component of combination regimens for severe CRAB infections (21). Cefoperazone-sulbactam is a widely used alternative formulation that enables high-dose sulbactam delivery. A recent multicenter, propensity score-matched study focusing on ICU patients with CRAB bloodstream infections (BSI) found that cefoperazone-sulbactam-containing combination therapy was associated with significantly lower 28-days all-cause mortality (30.0% vs. 50.0%) and clinical failure rates compared to regimens without it (22). These findings are supported by a 2024 meta-analysis of ten studies, which concluded that cefoperazone-sulbactam-based regimens were superior to non-cefoperazone-sulbactam-based regimens in reducing 30-days mortality and improving clinical outcomes in patients with multidrug-resistant *A. baumannii* infections (23). Furthermore, a large retrospective study on CRAB BSI of pulmonary origin suggested a survival benefit specifically associated with sulbactam-based therapy in this population (24). Evidence indicates that a higher daily sulbactam dose (≥8 g) is associated with improved microbiological and clinical efficacy in multidrug-resistant *A. baumannii* pneumonia (24).

Serial CSF mNGS enabled dynamic, quantitative monitoring of bacterial burden, allowing correlation of microbiological clearance with clinical improvement. The marked reduction in pathogen sequence reads (from 11,794 to 79) following initiation of intrathecal eravacycline provides compelling preliminary evidence of its intrathecal CNS efficacy. Notably, no adverse events directly attributable to intrathecal eravacycline, such as chemical meningitis, seizures, or significant neurotoxicity, were observed during treatment. This favorable safety profile suggests that, similar to intrathecal tigecycline, eravacycline may be a tolerable option for direct CNS administration under close monitoring.

The ultimate treatment success was achieved through therapeutic synergy of the final combination regimen, which integrated intrathecal eravacycline with high-dose intravenous cefoperazone-sulbactam and nebulized CMS. This multimodal strategy effectively targeted both the intracranial and pulmonary infection reservoirs while potentially mitigating systemic toxicity.

This case carries notable novelty, involving an adolescent female patient with simultaneous intracranial, pulmonary, and bloodstream CRAB infections, which presented an extremely challenging clinical scenario. Although systemic combination therapy contributed to the control of extracranial infections, the persistently high CSF pathogen load emphasized the need for direct intrathecal administration as a salvage strategy for refractory CRAB CNS infection. Based on pharmacological rationale and limited available clinical evidence, we recommend that such off-label use be employed with extreme caution and only when standard therapeutic options have been exhausted.

Our study has several limitations that warrant acknowledgment. First, this is a single case report without a control group, which limits definitive causal inference. Second, the independent contribution of intrathecal eravacycline cannot be fully isolated from other concurrent therapeutic interventions. Third, due to the lack of established pharmacokinetic data in pediatric or adolescent populations, the intrathecal eravacycline dosage was selected based on empirical clinical experience. Fourth, long-term neurological and infectious follow-up data remain limited. Fifth, the generalizability of this approach to other age groups or pathogen types is restricted. Accordingly, the conclusions drawn are hypothesis-generating and require validation in further clinical investigations. This expanded limitations section provides a transparent and balanced assessment of the constraints inherent in this study.

This is a particular case report, and the optimal dosage, stability, and long-term safety of intrathecal eravacycline remain undefined. Pharmacokinetic studies in CSF are urgently needed to establish target concentrations and dosing intervals. Nevertheless, in the context of rising antimicrobial resistance and the scarcity of new antibiotics with adequate CNS penetration, our experience supports the consideration of intrathecal eravacycline as a salvage option for extensively drug-resistant (XDR)-Gram-negative meningitis, particularly when guided by molecular diagnostics and multidisciplinary expertise.

## 4 Conclusion

This case demonstrates the successful use of intrathecal eravacycline combined with systemic therapy for the management of multifocal CRAB infections in a post-traumatic adolescent patient. Favorable clinical and microbiological outcomes, along with the absence of significant neurotoxicity, suggest that intrathecal eravacycline may represent a viable salvage option for CRAB CNS infections. Our clinical experience provides practical insights into the management of CRAB intracranial infections. Given the growing threat of CRAB antimicrobial resistance, further investigation is warranted regarding the CSF penetration of eravacycline, as well as its efficacy and safety in the treatment of CRAB CNS infections.

## Data Availability

The original contributions presented in this study are included in this article/supplementary material, further inquiries can be directed to the corresponding author.
